# Severe hypoglycemia in patients with liver cirrhosis and type 2 diabetes

**DOI:** 10.3389/fmed.2022.962337

**Published:** 2023-01-04

**Authors:** Fu-Shun Yen, Ming-Chih Hou, Jia-Sin Liu, Chih-Cheng Hsu, Chii-Min Hwu

**Affiliations:** ^1^Dr. Yen’s Clinic, Taoyuan, Taiwan; ^2^Department of Medicine, School of Medicine, Institute of Clinical Medicine, National Yang Ming Chiao Tung University, Hsinchu, Taiwan; ^3^Division of Gastroenterology and Hepatology, Department of Medicine, Taipei Veterans General Hospital, Taipei City, Taiwan; ^4^Institute of Population Health Sciences, National Health Research Institutes, Zhunan, Taiwan; ^5^Department of Health Services Administration, China Medical University, Taichung, Taiwan; ^6^Department of Family Medicine, Min-Sheng General Hospital, Taoyuan, Taiwan; ^7^National Center for Geriatrics and Welfare Research, National Health Research Institutes, Taipei City, Taiwan; ^8^Section of Endocrinology and Metabolism, Department of Medicine, Taipei Veterans General Hospital, Taipei City, Taiwan

**Keywords:** liver cirrhosis, all-cause mortality, chronic kidney disease, hypoglycemic agents, sulfonylurea

## Abstract

**Introduction:**

Advanced liver disease with massive liver damage may affect the metabolism of hypoglycemic agents and increase the risk of hypoglycemia. We conduct this research to compare the risk of severe hypoglycemia between patients with type 2 diabetes, with and without compensated liver cirrhosis.

**Methods:**

From Taiwan’s National Health Insurance Research Database, we identified persons with type 2 diabetes with cirrhosis (*n* = 18,209) and without cirrhosis (*n* = 538,510) from January 1, 2000, to December 31, 2010. Cox proportional hazards models were adopted to assess risks of all-cause mortality and severe hypoglycemia.

**Results:**

The mean follow-up period of this study was 3.7 years. The incidence rates of death during follow-up were 26.54 and 2.75 per 1,000 patient-years [aHR 7.63 (6.70–8.70)] for patients with cirrhosis and without cirrhosis, respectively. The incidence rates of severe hypoglycemia during follow-up were 0.53 and 0.14 per 1,000 patient-years [aHR 2.74 (1.52–4.92)] for patients with and without cirrhosis, respectively. The subgroup analysis of hypoglycemia risks in patients with and without cirrhosis disclosed no significant interaction for variables such as age, sex, chronic kidney disease, sulfonylurea use, number of oral antidiabetic drugs, insulin, b-blocker, and fibrate.

**Conclusion:**

This cohort study demonstrated that patients with type 2 diabetes and compensated cirrhosis showed a higher risk of mortality and severe hypoglycemia than those without liver cirrhosis.

## 1 Introduction

Hypoglycemia is the most feared consequence when caring for persons with diabetes mellitus (DM) (1). It can increase the risk of cardiovascular disease, cognition impairment (2–4), accidents, and mortality in patients with diabetes (1–3). Hypoglycemia can be unpleasant and cause sweating, palpitations, insomnia, and other psychosocial problems affecting the quality of life in patients (1–3). Patients experiencing hypoglycemia will have concerns with diabetic treatment and hesitate to follow therapeutic advice (1), thus compromising glucose control and increasing the risks of future diabetic complications.

During hypoglycemia, the pancreas decreases the secretion of insulin and increases the secretion of glucagon. Glucagon promotes hepatic glycogenolysis and gluconeogenesis (1, 3). As food is consumed, the hepatocytes absorb glucose from the intestine through glucose transporters, and convert it into glycogen stored in liver. Once the body has fasted for 2–6 h, the hepatocytes can break down the glycogen through glycogenolysis to release glucose for use. If body fasted for long time, the hepatocytes can use lactic acid, amino acid and glycerol to make glucose through the gluconeogenesis (3). The liver plays a critical role in gluconeogenesis, but in the case of prolonged starvation or massive liver injury, the kidney would substantially contribute to endogenous glucose production (3, 5). Studies showed that patients with alcoholic liver cirrhosis were prone to hypoglycemia (5), possibly due to malnutrition in patients with insufficient glycogen storage. Moreover, ethanol can affect the enzymes of gluconeogenesis and reduce hepatic glucose production (5). Liver cirrhosis caused by non-alcoholic fatty liver disease, and hepatitis B or C viral infection may also tend to induce hypoglycemia, but without clear evidence (6). If patients with liver cirrhosis receive hypoglycemic agents, the portosystemic shunt of the liver increases the blood concentration of these drugs (7). Cirrhosis associated with massive liver damage and prominent hepatic dysfunction may affect the metabolism and clearance of hypoglycemic agents and increase the risk of hypoglycemia (8).

As there is currently no conclusive empirical evidence indicating liver cirrhosis (caused by viral hepatitis or non-alcoholic fatty liver disease) has a higher risk of hypoglycemia, we conduct this cohort study to determine the risks of severe hypoglycemia and mortality between persons with type 2 diabetes (T2D) with and without compensated liver cirrhosis. We also investigate whether different hypoglycemic agents have a dissimilar impact on hypoglycemic risk in these patients. Patients with cirrhosis have a significantly higher mortality risk than those without cirrhosis (9), and if patients died early during the study period due to cirrhosis, this may affect the detection of hypoglycemia. Therefore, in this study, we also compared the risk of death between diabetic patients with and without cirrhosis; and taking death as a competing risk, we used competing risk analyses to compare the risk of hypoglycemia in these patients.

## 2 Materials and methods

### 2.1 Data source

Taiwan’s National Health Insurance (NHI) program was constructed in 1995. It took care of approximately 99% of the 23 million people in Taiwan over the years (10). The health care information of insurers is recorded in the NHI research dataset (NHIRD), which includes age, sex, residence, premium levels, procedures, medications, and diagnostic codes based on the International Classification of Diseases 9th revision Clinical Modification (ICD-9-CM). We identified study subjects from the NHIRD. This study was approved by The Institutional Review Board of the National Health Research Institutes (EC1060704-E). As all information from patients and care providers was scrambled and encrypted before release, we were granted to waive the informed consents from patients by the Institutional Review Board.

### 2.2 Experimental design

**FIGURE 1:**
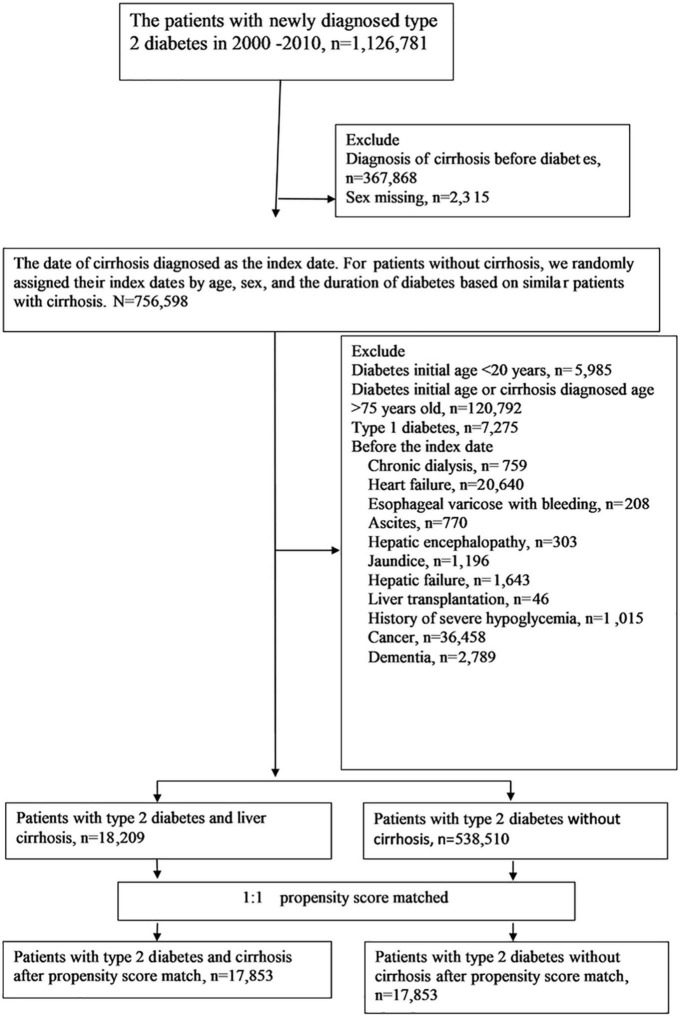
Flowchart of the selection of cohorts from the National Health Insurance Research Database.

We selected patients with newly diagnosed type 2 diabetes from January 1, 2000, to December 31, 2010 (Figure 1). Type 2 diabetes was diagnosed using the ICD-9-CM code 250.xx for at least two outpatient visits in 1 year or one hospitalization, and under hypoglycemic agents treatment. Liver cirrhosis was defined as a diagnosis of ICD-9-CM codes 571.5, 571.2, or 571.6 for at least two outpatient claims in 1 year or one hospitalization. The algorithm for the definitions of T2D and liver cirrhosis based on ICD-9 coding has been validated by previous medical studies (11, 12), with the accuracy of T2D and cirrhosis diagnosis being 74.6 and 82.6%, respectively. Patients with cirrhosis and variceal bleeding (456.0, 456.2), hepatic encephalopathy (572.2), or ascites (789.59, 789.5) were defined as decompensated cirrhosis (13); patients without such complications were defined as compensated cirrhosis. We excluded patients with the diagnosis of cirrhosis before T2D, sex missing, age younger than 20 or older than 75 years, diagnosis of type 1 diabetes (250.1), diagnoses of chronic dialysis (V56.0, V56.8, V45.1), heart failure (428), decompensated cirrhosis [including esophageal varicose with bleeding (456.0, 456.2), hepatic encephalopathy (572.2), and ascites (789.59, 789.5)], jaundice (782.4), hepatic failure (570, 572.2, 572.4, 572.8), liver transplantation (V42.7,996.82, procedure code 50.5), cancers (140–239, except 210–229), dementia (294, 331.0), and history of severe hypoglycemia before the index date.

### 2.3 Study procedures

We defined the first date of a new diagnosis of liver cirrhosis as the index date. For patients without cirrhosis, we randomly assigned their index dates by age, sex, and the duration of diabetes based on matched patients with cirrhosis. The potentially confounding variables of this study included age, sex, smoking (305.1, 649.0, V15.82), alcoholic disease (291, 303, 305.0, 571.0, 570.1, 571.3, V11.3, V79.1), body weight [severe obesity (278.01, 649.2, V45.86, V85.4), obesity (278.00, 649.1, V77.8, V85.3), overweight (278.02, 783.1, V85.2)], hepatitis B virus (HBV) infection (070.2, 070.3, V02.61), HBV therapy (lamivudine, adefovir dipivoxil, tenofovir disoproxil, entecavir, and telbivudine), hepatitis C virus (HCV) infection (070.41, 070.44, 070.51, 070.54, 070.70, 070.71, V02.62), HCV therapy (interferons), chronic obstructive pulmonary disease (COPD, 491, 492, 496), chronic kidney disease (CKD; 403.01, 403.11, 403.91, 404.02, 404.03, 404.12, 404.13, 404.92, 404.93, 581–588 and A350), and Charlson Comorbidity Index (CCI) scores (14) (comorbidities diagnosed within 1 year of the index date), prescriptions, such as metformin, sulfonylureas, glinides, thiazolidinedione, dipeptidyl peptidase-4 inhibitors (DPP-4i), basal insulin, premixed insulin, short-acting insulin, angiotensin receptor blockers (ARB), angiotensin-converting enzyme inhibitor (ACEi), β-blockers, calcium-channel blockers, diuretics, statins, fibrate, aspirin, and the enrolled research year of 2000–2004, 2005–2008, and 2009–2010. We used the Diabetes Complications Severity Index (DCSI) score (15), duration of type 2 diabetes, and the number of oral hypoglycemic agents to reflect the severity of diabetes in this study.

### 2.4 Main outcomes

All-cause mortality and severe hypoglycemia were the main study outcomes. Mortality was defined as discharge from hospital with ascertained death (the discharge day was defined as the death day) or stop of the NHI coverage after hospital discharge due to a critical illness without further healthcare usage in the NHI records for longer than 1 year (the end of NHI coverage was set as the death day). Severe hypoglycemia was defined in patients requiring a visit to emergency department or hospitalization for hypoglycemia (251.0x, 251.1x, or 251.2x). We assessed the incidence rates of mortality and severe hypoglycemia between the cirrhosis and non-cirrhosis groups during the follow-up period.

### 2.5 Statistical analysis

We utilized propensity score matching to decrease the imbalance between patients with and without liver cirrhosis (16). Using non-parsimonious multivariable logistic regression, we estimated the propensity score for every patient with the diagnosis of cirrhosis as the dependent variable; and clinically related variables in the analysis as independent variables (Table 1). The nearest-neighbor algorithm was utilized to select matched pairs, with the standardized differences less than 0.05 as perfect.

**TABLE 1:** Basic characteristics of pre-matched and post-matched data in patients with type 2 diabetes with and without liver cirrhosis.

	Pre-propensity-score matched				Post-propensity-score matched			
	**With cirrhosis**	**Without cirrhosis**	***P*** **-value**	**SID**	**With cirrhosis**	**Without cirrhosis**	***P*** **-value**	**SID**
*n*	18,209	5,38,510			17,853	17,853		
**Age group**								
20–39	917 (5)	40,286 (7.5)	<0.001	0.101	883 (4.9)	936 (5.2)	0.20	0.014
40–64	12,614 (69.3)	3,72,795 (69.2)	0.89	0.001	12,359 (69.2)	12,219 (68.4)	0.11	0.017
65–74	4,678 (25.7)	1,25,429 (23.3)	<0.001	0.056	4,611 (25.8)	4,698 (26.3)	0.29	0.011
Mean (SD)	56.7 (10.1)	55.8 (10.6)	<0.001	0.086	56.7 (10.1)	56.5 (10.4)	0.021	0.024
Gender			<0.001	0.307			0.038	0.022
Male	12,799 (70.3)	2,99,545 (55.6)			12,489 (70)	12,308 (68.9)		
Female	5,410 (29.7)	2,38,965 (44.4)			5,364 (30)	5,545 (31.1)		
**Comorbidity**								
Smoking	46 (0.3)	956 (0.2)	0.019	0.016	44 (0.2)	32 (0.2)	0.17	0.015
Alcohol	1,544 (8.5)	5,357 (1)	<0.001	0.358	1,420 (8)	1,479 (8.3)	0.25	0.012
Overweight	3 (< 0.1)	377 (0.1)	0.007	0.026	3 (< 0.1)	4 (< 0.1)	0.71	0.004
Obesity	121 (0.7)	6,020 (1.1)	<0.001	0.048	120 (0.7)	141 (0.8)	0.19	0.014
Severe obesity	17 (0.1)	567 (0.1)	0.62	0.004	15 (0.1)	18 (0.1)	0.6	0.006
HBV	2,236 (12.3)	15,714 (2.9)	<0.001	0.359	2,089 (11.7)	2,250 (12.6)	0.009	0.028
HBV therapy	431 (2.4)	248 (0)	<0.001	0.214	293 (1.6)	221 (1.2)	0.001	0.034
HCV	2,285 (12.5)	7,678 (1.4)	<0.001	0.447	2,134 (12)	2,317 (13)	0.003	0.031
HCV therapy	253 (1.4)	229 (0)	<0.001	0.160	206 (1.2)	178 (1)	0.15	0.015
CKD	1,425 (7.8)	28,023 (5.2)	<0.001	0.106	1,393 (7.8)	1,400 (7.8)	0.89	0.001
COPD	1,689 (9.3)	36,911 (6.9)	<0.001	0.089	1,661 (9.3)	1,628 (9.1)	0.55	0.006
**CCI scores**								
Mean (SD)	3.4 (2.0)	2.3 (1.5)	<0.001	0.653	3.4 (2.0)	3.4 (2.1)	<0.001	0.016
**DCSI**								
0	10,739 (59)	3,21,931 (59.8)	0.029	0.016	10,520 (58.9)	10,092 (56.5)	<0.001	0.049
1	4,001 (22)	1,24,532 (23.1)	0.001	0.028	3,919 (22)	4,303 (24.1)	<0.001	0.051
≧2	3,469 (19.1)	92,047 (17.1)	<0.001	0.051	3,414 (19.1)	3,458 (19.4)	0.55	0.006
Duration of diabetes, years	2.6 (2.4)	2.8 (0.7)	<0.001	0.113	2.7 (2.4)	2.7 (0.7)	0.002	0.034
**Hypoglycemic agents**								
Metformin	8,823 (48.5)	2,27,133 (42.2)	<0.001	0.126	8,620 (48.3)	8,709 (48.8)	0.35	0.010
Sulfonylurea	10,675 (58.6)	2,44,924 (45.5)	<0.001	0.265	10,423 (58.4)	10,382 (58.2)	0.66	0.005
TZD	1,174 (6.4)	34,802 (6.5)	0.93	0.001	1,153 (6.5)	1,180 (6.6)	0.56	0.006
DPP-4i	226 (1.2)	4,264 (0.8)	<0.001	0.045	213 (1.2)	217 (1.2)	0.85	0.002
**Number of OHA**								
0	5,373 (29.5)	2,29,686 (42.7)	<0.001	0.276	5,311 (29.7)	5,674 (31.8)	<0.001	0.044
1	4,620 (25.4)	1,09,946 (20.4)	<0.001	0.118	4,525 (25.3)	3,847 (21.5)	<0.001	0.090
2	6,190 (34)	1,54,807 (28.7)	<0.001	0.113	6,059 (33.9)	6,209 (34.8)	0.09	0.018
3	1,647 (9.0)	37,967 (7.1)	<0.001	0.073	1,595 (8.9)	1,743 (9.8)	0.007	0.028
≧4	379 (2.1)	6,104 (1.1)	<0.001	0.075	363 (2.0)	380 (2.1)	0.53	0.007
**Insulin**								
Basal	150 (0.8)	1,914 (0.4)	<0.001	0.061	146 (0.8)	137 (0.8)	0.59	0.006
Premixed	387 (2.1)	2,639 (0.5)	<0.001	0.144	355 (2.0)	351 (2.0)	0.88	0.002
Fast-acting	696 (3.8)	2,476 (0.5)	<0.001	0.234	611 (3.4)	624 (3.5)	0.71	0.004
**Cardiovascular drugs**								
ACEi/ARBs	5,046 (27.7)	1,48,269 (27.5)	0.60	0.004	4,971 (27.8)	5,339 (29.9)	<0.001	0.045
Beta-blocker	2,734 (15.0)	83,131 (15.4)	0.12	0.012	2,696 (15.1)	2,900 (16.2)	0.003	0.031
CCB	5,212 (28.6)	1,40,622 (26.1)	<0.001	0.056	5,130 (28.7)	5,448 (30.5)	0.001	0.039
Diuretics	6,116 (33.6)	72,618 (13.5)	<0.001	0.488	5,884 (33)	5,977 (33.5)	0.30	0.011
Statin	1966 (10.8)	1,01,989 (18.9)	<0.001	0.230	1,948 (10.9)	2,100 (11.8)	0.011	0.027
Fibrate	1,490 (8.2)	46,611 (8.7)	0.026	0.017	1,474 (8.3)	1,552 (8.7)	0.14	0.016
Aspirin	2,396 (13.2)	78,947 (14.7)	<0.001	0.043	2,370 (13.3)	2,470 (13.8)	0.12	0.016
**Enrolled research year**								
2000–2004	5,341 (29.3)	1,13,897 (21.2)	<0.001	0.189	5,249 (29.4)	5,221 (29.2)	0.74	0.003
2005–2008	8,097 (44.5)	2,70,198 (50.2)	<0.001	0.115	7,960 (44.6)	8,054 (45.1)	0.32	0.011
2009–2010	4,771 (26.2)	1,54,415 (28.7)	<0.001	0.055	4,644 (26.0)	4,578 (25.6)	0.42	0.008
Propensity score	0.13681 (0.1846)	0.02931 (0.0454)	<0.001	0.799	0.12322 (0.1595)	0.12279 (0.1581)	0.80	0.003

SID, standardized differences; SD, standard deviation; HBV, Hepatitis B virus infection; HCV, Hepatitis C virus infection; CKD, chronic kidney disease; COPD, chronic obstructive pulmonary disease; CCI, Charlson Comorbidity Index; DCSI, diabetes complications severity index; TZD, thiazolidinedione; DDP-4i, dipeptidyl peptidase-4 inhibitor; OHA, oral hypoglycemic agents; ACEi, angiotensin-converting enzyme inhibitor; ARBs, angiotensin receptor blockers; CCB, calcium channel blocker.

Multivariable-adjusted Cox proportional hazards models were used to determine the outcomes between patients with and without cirrhosis. The results were shown as hazard ratios (HR) with a 95% confidence interval (CI). Owning to the competing risk of death might confound the estimate of risk for severe hypoglycemia, we adopted the competing risk analyses for adjustment (Table 2) (17). To calculate the risk of death, we censored patients at the time of mortality or the end of the research, whichever came first. For hypoglycemic outcomes, we reviewed patients on the day of hypoglycemia or the end of follow-up on December 31, 2010.

**TABLE 2:** The risk of patients with diabetes and with or without cirrhosis in multivariate Cox’s regression analysis.

	With cirrhosis		Without cirrhosis		Crude model		Adjusted model		After propensity matched adjusted model	
	***n***	**IR**	***n***	**IR**	**Hazard ratio (95% confidence interval)**	***P*** **-Value**	**Hazard ratio (95% confidence interval)**	***P*** **-value**	**Hazard ratio (95% confidence interval)**	***P*** **-value**
All-cause mortality	1,924	26.54	5,523	2.75	9.52 (9.03–10.0)	<0.001	8.03 (7.57–8.52)	<0.001	7.63 (6.70–8.70)	<0.001
Hypoglycemia	38	0.53	284	0.14	3.70 (2.64–5.20)	<0.001	3.17 (2.19–4.60)	<0.001	2.74 (1.52–4.92)	<0.001
Hypoglycemia^a^							2.96 (2.04–4.28)	<0.001	2.56 (1.42–4.63)	<0.001
Hypoglycemia^b^							3.19 (2.20–4.60)	<0.001	2.74 (1.52–4.93)	<0.001

*n*, case number; IR, incidence rate, per 1,000 person-years; Adjusted model, adjusted age, sex, smoking, alcohol, overweight, obesity, severe obesity, HBV infection and therapy, HCV infection and therapy, CKD, COPD, CCI score, DCSI, duration of diabetes, metformin, sulfonylurea, TZD, DDP-4i, insulin basal, insulin premixed, insulin basal and bolus, number of oral hypoglycemic agents, ACEI/ARB, beta-blocker, CCB, diuretics, statin, fibrate, aspirin, and enrolled research year.

^a^Taking death as a competing risk for sub-distribution competing risk analysis of hypoglycemia.

^b^Taking death as a competing risk for cause-special competing risk analysis of hypoglycemia.

We estimated the cumulative incidence of all-cause death and severe hypoglycemia over time between patients with and without cirrhosis using the Kaplan-Meier method; we determined whether significant differences existed between the study and comparison groups by the log-rank test. We did a subgroup analysis for the risk of severe hypoglycemia and likelihood-ratio test to identify significant interactions between cirrhosis and age, sex, CKD, sulfonylurea use, glibenclamide, glipizide, gliclazide, glimepiride, number of oral hypoglycemic agents, insulin, β-blocker, and fibrate. A two-sided *p*-value of less than 0.05 was considered as statistically significant. SAS version 9.4 and Stata SE version 15.1 were utilized for the analyses of this study.

## 3 Results

From January 1, 2000, to December 31, 2010, a total of 18,209 patients were under the diagnosis of type 2 diabetes and compensated cirrhosis, while 538,510 patients were under the diagnosis of T2D and without cirrhosis (Figure 1). Before matching, some differences were acknowledged between the study and comparison groups (Table 1). After propensity score matching, 17,853 pairs of T2D patients with and without cirrhosis were chose. The matched pairs of patients were similar for all variables. The mean age of the matched cohort was 56.6 years; the mean duration of diabetes was 2.7 years; the prevalence rates of HBV and HCV infections were 13.6 and 13.6%, respectively. The mean follow-up time (SD) was 3.7 (2.4) years in this study.

In the pre-matched cohort of T2D with and without liver cirrhosis, 1,924 (10.57%) patients with cirrhosis and 5,523 (1.03%) patients without cirrhosis died during the tracked time (incidence rate 26.54 vs. 2.75 per 1,000 person-years). The adjusted multivariable models revealed that cirrhotic patients had significantly increased mortality risk; the pre-matched aHR was 8.03 (7.57–8.52), and the post-matched aHR was 7.63 (6.70–8.70; Table 2).

In the pre-matched cohort of T2D with and without liver cirrhosis, 38 (0.21%) patients with cirrhosis and 284 (0.05%) patients without cirrhosis had severe hypoglycemia during the tracked time (incidence rate 0.53 vs. 0.14 per 1,000 person-years). The adjusted multivariable models revealed that cirrhotic patients had a significantly increased risk of severe hypoglycemia; the pre-matched aHR was 3.17 (2.19–4.60), and the post-matched aHR was 2.74 (1.52–4.92; Table 2). Taking death as a competing risk, the sub-distribution competing risk analysis showed the post-matched aHR of hypoglycemia was 2.56 (1.42–4.63); for cause-special competing risk analysis, the aHR of hypoglycemia was 2.74 (1.52–4.93; Table 2).

**FIGURE 2:**
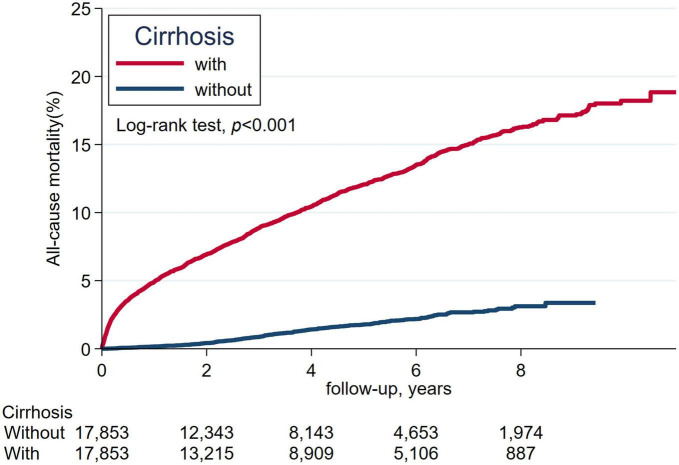
The cumulative incidence of all-cause mortality between patients with cirrhosis and without cirrhosis.

**FIGURE 3:**
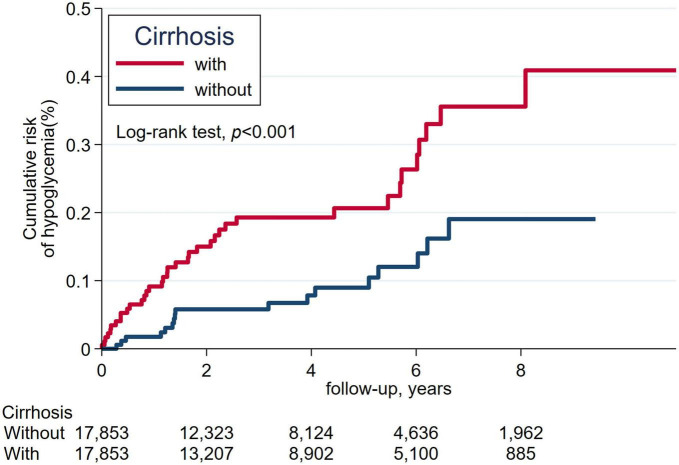
The cumulative incidence of severe hypoglycemia between patients with cirrhosis and without cirrhosis.

The cumulative incidence of mortality in matched patients with cirrhosis exhibited a significantly increased risk than in patients without cirrhosis (Figure 2). The cumulative incidence of severe hypoglycemia in matched patients with cirrhosis displayed a significantly increased risk than in patients without cirrhosis (Figure 3).

**FIGURE 4:**
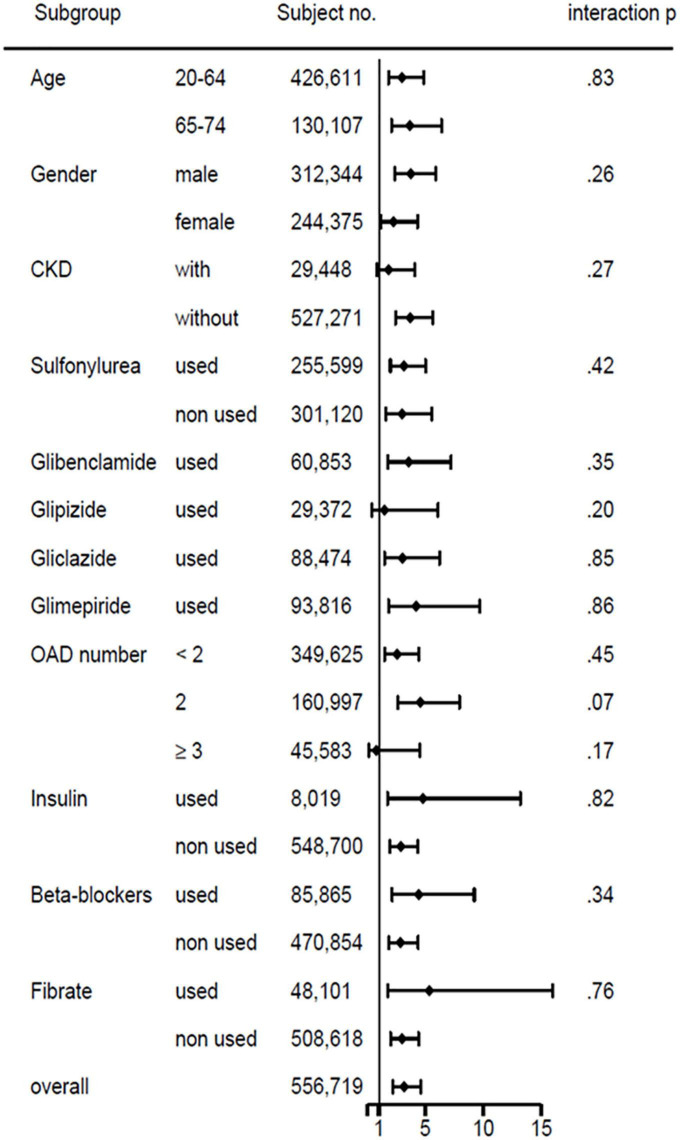
Subgroup analysis of basic demographics, comorbidity, and medications on hypoglycemia risks in patients with type 2 diabetes with versus without liver cirrhosis.

The forest plot of subgroup analysis of hypoglycemia risks in patients with cirrhosis versus without cirrhosis showed no significant interaction in the risk factors of age, sex, CKD, sulfonylurea, glibenclamide, glipizide, gliclazide, glimepiride, number of oral hypoglycemic agents, insulin, β-blocker, and fibrate (Figure 4).

## 4 Discussion

The present study demonstrated that T2D persons with compensated cirrhosis had higher risks of mortality and severe hypoglycemia than those without liver cirrhosis. Moreover, patients with liver cirrhosis had an increased risk of hypoglycemia, regardless of age, sex, chronic kidney disease, insulin, sulfonylureas, and different types of sulfonylurea, number of oral hypoglycemic agents, β-blocker, and fibrate use.

Patients with compensated liver cirrhosis are at a 4.7 times increased risk of mortality than the general population (9). Moreover, cirrhosis can exacerbate insulin resistance and disrupt carbohydrate metabolism in patients with T2D, resulting in increased complications and premature mortality (6). Our study indicated that patients with T2D and compensated liver cirrhosis had a 7.6 times higher risk of mortality than those without liver cirrhosis. On the other hand, diabetes can also increase the risk of death in patients with compensated liver cirrhosis (7, 18), possibly through the progression of liver cirrhosis and increased bacterial infection (7, 18).

Episodes of severe hypoglycemia alter the mental state of the patient. The individual is unable to take care of himself and requires the assistance of others (19). The incidence rate of severe hypoglycemia in persons with type 2 diabetes is approximately 0.1–0.7% (19) or 0.05 episodes per 100 patient-years (20); it is 0.8 (or 0.9 events per 100 patients-years) (21) to 7% (10 episodes per 100 patient-years) in T2D patients on sulfonylurea (19, 20) and 1.2–7.3% (or 11.8 episodes per 100 patient-years) (20) in patients with T2D on insulin (19, 21). The incidence rate of severe hypoglycemia in persons with type 1 diabetes on insulin is 7.1% (21). Hypoglycemia has long been noted in association with chronic alcohol use and inadequate dietary intake (5). One case report of fulminant hepatitis has presented with severe hypoglycemia (22). In a case series of viral hepatitis, eight of the 15 patients (53.3%) showed plasma glucose levels below 60 mg/dl but no symptoms of hypoglycemia (23). A retrospective analysis of 87 cirrhotic patients reported that 11 patients (12.6%) had decreased glucose levels (<70 mg/dl) or documented hypoglycemia (24).

To our knowledge, our research is the first aiming to compare the risk of severe hypoglycemia between T2D patients with and without compensated liver cirrhosis. It showed that the incidence of severe hypoglycemia was 0.53 per 1,000 person-year in persons with T2D and cirrhosis. Patients with T2D and compensated liver cirrhosis had a 2.7 times higher risk of hypoglycemia than those without liver cirrhosis. Reports show that age, renal impairment, insulin use, sulfonylurea use, β-blocker, or fibrate are associated with a higher risk of hypoglycemia (1, 19, 20). However, the subgroup analysis of hypoglycemia risk in our patients with compensated cirrhosis versus without cirrhosis disclosed no significant interaction existed in these risk factors.

The increased risk of severe hypoglycemia in persons with compensated liver cirrhosis may arise from multiple roots. First, the liver plays a vital role in bolstering glucose homeostasis, hypoglycemia will not happen until most of the liver is injured or removed (5). Cirrhosis with widespread extinction and collapse of hepatic parenchyma may reduce glycogenolysis, gluconeogenesis, and glucose output (5). Second, the impaired ability of the cirrhotic liver to synthesize or store glycogen may reduce glucose release during starvation or prolonged fasting (25). Third, gluconeogenesis is the primary process that affords glucose production during hypoglycemic status, and a limitation of necessary substrates (such as amino acids, lactate, and glycerol) for gluconeogenesis in patients with cirrhosis and malnutrition may increase hypoglycemia risk (3). Fourth, the markedly impaired glucose response to glucagon in patients with liver cirrhosis affects glucose release during hypoglycemia (26). Fifth, the portosystemic and intrahepatic shunting, reduced insulin extraction, and decreased sulfonylurea metabolism due to cirrhosis may raise the risk of hypoglycemia in patients taking insulin or insulin secretagogues (27).

Our study has some advantages. First, this is a nationwide population-based study. We non-selectively identified patients from the National Health Insurance Research Database to avoid selection bias. Second, this study has a large sample size and spans 10 years (from 2000 to 2010), with enough power to investigate the real-world risk of severe hypoglycemia in patients with liver cirrhosis.

Nevertheless, this research is subject to some disadvantages. First, we defined severe hypoglycemia based on patients requiring emergency department visits or hospitalization due to hypoglycemia, which may underestimate the incidence of severe hypoglycemia in this study. However, this definition was undifferentially applied in the study and control groups, and hence, may not influence the comparison of endpoints. Second, this dataset lacked information on biochemical test results; therefore, we could not count the Child-Pugh Class or Model for End-stage Liver Disease scores to determine the severity of liver cirrhosis; we also can’t ensure the treated condition of T2D due to the lack of hemoglobin A1C, and glucose levels. Alternately, we used the clinical diagnosis to classify patients into compensated and decompensated cirrhosis. Some patients with minimal hepatic encephalopathy or mild-moderate ascites may have escaped detection in this study with less exclusion of decompensated cirrhosis, and affecting study results. However, we included gender, age, comorbidities, CCI, DCSI scores, and prescriptions to maximally balance the condition between the study and comparison groups and increase their comparability. Third, the national health dataset did not provide complete information on the dietary pattern, alcohol consumption, physical activity, and medication compliance, influencing measured outcomes. Fourth, this research was conducted among Taiwanese subjects, and the results may not apply to other ethnic groups. Finally, a cohort study is usually subject to some unknown or unobserved confounding factors, and a prospective randomized control study is warranted to verify our results.

## 5 Conclusion

Liver cirrhosis is the ultimate stage of hepatic injury. Its severity may vary from person to person, and the treatment of T2D in these patients is usually complex. Our study showed that persons with T2D and compensated liver cirrhosis had higher mortality risk and severe hypoglycemia. We recommend regular and nutritious diets, alcohol abstinence, antiviral therapy for hepatitis B or C infections, and frequent blood glucose monitoring for these patients.

## Data availability statement

The original contributions presented in this study are included in the article/Supplementary material, further inquiries can be directed to the corresponding authors.

## Ethics statement

This study was approved by the Institutional Review Board of the National Health Research Institutes (EC1060704-E). Written informed consent for participation was not required for this study in accordance with the national legislation and the institutional requirements.

## Author contributions

F-SY and C-CH contributed equally in this study and participated in manuscript preparation. F-SY, M-CH, C-MH, and C-CH conceived and designed the study. J-SL, M-CH, and C-CH contributed to the data acquisition. F-SY and J-SL participated in statistical analysis and interpretation of data. C-MH obtained funding. J-SL, M-CH, and C-MH contributed to the administrative, technical, or material support. M-CH and C-MH supervised the study. All authors participated in the critical revision of the manuscript for important intellectual content.

## References

[1] Oyer DS. The science of hypoglycemia in patients with diabetes. *Curr Diabetes Rev.* (2013) 9:195–208. 10.2174/15733998113099990059 23506375

[2] International Hypoglycaemia Study Group. Hypoglycaemia, cardiovascular disease, and mortality in diabetes: epidemiology, pathogenesis, and management. *Lancet Diabetes Endocrinol.* (2019) 7:385–96. 10.1016/S2213-8587(18)30315-2 30926258

[3] Cryer P. *Hypoglycemia in Diabetes: Pathophysiology, Prevalence and Prevention.* Alexandria, VA: American Diabetes Association (2012). p. 37–125.

[4] Warren RE Frier BM. Hypoglycaemia and cognitive function. *Diabetes Obes Metab.* (2005) 7:493–503. 10.1111/j.1463-1326.2004.00421.x 16050942

[5] Arky RA. Hypoglycemia associated with liver disease and ethanol. *Endocrinol Metab Clin North Am.* (1989) 18:75–90. 10.1016/S0889-8529(18)30389-X 2645130

[6] Tolman KG Fonseca V Dalpiaz A Tan MH. Spectrum of liver disease in type 2 diabetes and management of patients with diabetes and liver disease. *Diabetes Care.* (2007) 30:734–43. 10.2337/dc06-1539 17327353

[7] Garcia-Compean D Jaquez-Quintana JO Gonzalez-Gonzalez JA Maldonado-Garzae H. Liver cirrhosis and diabetes: risk factors, pathophysiology, clinical implications and management. *World J Gastroenterol.* (2009) 15:280–8. 10.3748/wjg.15.280 19140227 PMC2653324

[8] Gangopadhyay KK Singh P. Consensus statement on dose modifications of antidiabetic agents in patients with hepatic impairment. *Indian J Endocrinol Metab.* (2007) 21:341–54. 10.4103/ijem.IJEM_512_16 PMC5367241 28459036

[9] Cheng TM. Taiwan’s new national health insurance program: genesis and experience so far. *Health Aff.* (2003) 22:61–76. 10.1377/hlthaff.22.3.61 12757273

[10] Lin CC Lai MS Syu CY Chang SC Tseng FY. Accuracy of diabetes diagnosis in health insurance claims data in Taiwan. *J Formos Med Assoc.* (2005) 104:157–63. 15818428

[11] Nehra MS Ma Y Clark C Rockey DC Singal AG. Use of administrative claims data for identifying patients with cirrhosis. *J Clin Gastroenterol.* (2013) 47:e50–4. 10.1097/MCG.0b013e3182688d2f 23090041 PMC3556340

[12] Mukerji AN Patel V Jain A. Improving survival in decompensated cirrhosis. *Int J Hepatol.* (2012) 2012:318627. 10.1155/2012/318627 22811919 PMC3395145

[13] Meduru P Helmer D Rajan M Tseng CL Pogach L Sambamoorthi U. Chronic illness with complexity: implications for performance measurement of optimal glycemic control. *J Gen Intern Med.* (2007) 22:408–18. 10.1007/s11606-007-0310-5 18026810 PMC2150612

[14] Young BA Lin E Von Korff M Simon G Ciechanowski P Ludman EJ Diabetes complications severity index and risk of mortality, hospitalization, and health care utilization. *Am J Manag Care.* (2008) 14:15–23. 18197741 PMC3810070

[15] D’Agostino RB Jr. Propensity score methods for bias reduction in the comparison of a treatment to a non-randomized control group. *Stat Med.* (1998) 17:2265–81. 9802183 10.1002/(sici)1097-0258(19981015)17:19<2265::aid-sim918>3.0.co;2-b

[16] Lau B Cole SR Gange SJ. Competing risk regression models for epidemiologic data. *Am J Epidemiol.* (2009) 170:244–56. 10.1093/aje/kwp107 19494242 PMC2732996

[17] Fleming KM Aithal GP Card TR West J. All-cause mortality in people with cirrhosis compared with the general population: a population-based cohort study. *Liver Int.* (2012) 32:79–84. 10.1111/j.1478-3231.2011.02517.x 21745279

[18] Quintana JO García-Compean D González JA Pérez JZV González FJL Espinosa LEM The impact of diabetes mellitus in mortality of patients with compensated liver cirrhosis-a prospective study. *Ann Hepatol.* (2011) 10:56–62. 21301011

[19] Amiel SA Dixon T Mann R Jameson K. Hypoglycaemia in type 2 diabetes. *Diabetic Med.* (2008) 25:245–54. 10.1111/j.1464-5491.2007.02341.x 18215172 PMC2327221

[20] Inkster B Zammitt NN Frier BM. Drug-induced hypoglycaemia in type 2 diabetes. *Expert Opin Drug Saf.* (2012) 11:597–614. 10.1517/14740338.2012.694424 22690846

[21] Leese GP Wang J Broomhall J Kelly P Marsden A Morrison W DARTS/MEMO Collaboration. Frequency of severe hypoglycemia requiring emergency treatment in type 1 and type 2 diabetes. *Diabetes Care.* (2003) 26:1176–80. 10.2337/diacare.26.4.1176 12663593

[22] Samson RI Trey C Timme AH Saunders SJ. Fulminating hepatitis with recurrent hypoglycemia and hemorrhage. *Gastroenterology.* (1967) 53:291–300. 10.1016/S0016-5085(19)34237-4

[23] Felig P Brown WV Levine RA Klatskin G. Glucose homeostasis in viral hepatitis. *N Engl J Med.* (1970) 283:1436–40. 10.1056/NEJM197012242832604 5481777

[24] Gundling F Seidl H Strassen I Haller B Siegmund T Umgelter A Clinical manifestations and treatment options in patients with cirrhosis and diabetes mellitus. *Digestion.* (2013) 87:75–84. 10.1159/000343458 23306648

[25] British Medical Journal,. Hepatic hypoglycaemia. *Br Med J.* (1971) 2:416–7. PMC1796172 5575997

[26] Yeung RT Wang CC. A study of carbohydrate metabolism in postnecrotic cirrhosis liver. *Gut.* (1974) 15:907–12. 10.1136/gut.15.11.907 4455570 PMC1413049

[27] Scheen AJ. Pharmacokinetic and toxicological considerations for the treatment of diabetes in patients with liver disease. *Expert Opin Drug Metab Toxicol.* (2014) 10:839–57. 10.1517/17425255.2014.902444 24669954

