# Supplementary material for: Severe hypoglycemia in patients with liver cirrhosis and type 2 diabetes
Source: Front Med (Lausanne). 2023 Jan 4;9:962337. doi: 10.3389/fmed.2022.962337 (PMC9845885; doi:10.3389/fmed.2022.962337)
Supplement: Supplementary file 1 [file Table_1.DOCX]

Supplementary Material

## Supplementary Tables

**Supplementary Table 1.** The risk of patients with diabetes and with or without cirrhosis in multivariate Cox’s regression analysis (excluding patients with HBV or HCV infection).

|  | **With cirrhosis** | | **Without cirrhosis** | | **Crude model** | **Adjusted model** | **After propensity matched adjusted model** |
| --- | --- | --- | --- | --- | --- | --- | --- |
|  | **n** | **IR** | **n** | **IR** | **cHR (95%CI)** | **aHR (95%CI)** | **aHR (95%CI)** |
| All-cause mortality | 1606 | 27.49 | 5489 | 2.83 | 9.55 (9.02-10.1) | 7.84 (7.38-8.34) | 7.41 (6.43-8.55) |
| Hypoglycemia | 43 | 0.74 | 306 | 0.16 | 4.64 (3.37-6.40) | 3.64 (2.58-5.15) | 2.53 (1.39-4.60) |

*n, case number; IR, incidence rate, per 1.000 person-years; Adjusted model adjusted age, sex, smoking, alcohol, overweight, obesity, severe obesity, CKD, COPD, CCI score, DCSI, duration of diabetes, metformin, sulfonylurea, TZD, DDP-4i, insulin basal, insulin premixed, insulin basal and bolus, number of oral hypoglycemic agents, ACEI/ARB, beta-blocker, CCB, diuretics, statin, fibrate, aspirin, and enrolled research year*.

**Supplementary Table 2.** Subgroups of DCSI and risks of mortality and hypoglycemia in multivariate Cox’s regression analysis.

|  | **With cirrhosis** | | **Without cirrhosis** | | **Adjusted model** |
| --- | --- | --- | --- | --- | --- |
|  | **n** | **IR** | **n** | **IR** | **aHR (95%CI)** |
| All-cause mortality |  |  |  |  |  |
| DCSI=0 | 1143 | 25.9 | 2136 | 1.81 | 12.3 (11.3-13.4) |
| DCSI=1 | 373 | 23.33 | 1147 | 2.4 | 7.65 (6.69-8.73) |
| DCSI≧2 | 436 | 33.64 | 2358 | 6.55 | 4.33 (3.87-4.84) |
| Hypoglycemia (+) | 10 | 35.19 | 25 | 14.68 | 2.91 (1.10-7.67) |
| Hypoglycemia (-) | 1942 | 26.68 | 5616 | 2.79 | 7.94 (7.49-8.42) |
| Hypoglycemia |  |  |  |  |  |
| DCSI=0 | 19 | 0.43 | 102 | 0.09 | 4.73 (2.69-8.32) |
| DCSI=1 | 13 | 0.82 | 64 | 0.13 | 4.92 (2.46-9.84) |
| DCSI≧2 | 17 | 1.32 | 146 | 0.41 | 2.60 (1.52-4.47) |

*n, case number; IR, incidence rate, per 1.000 person-years; Adjusted model adjusted age, sex, smoking, alcohol, overweight, obesity, severe obesity, CKD, COPD, CCI score, DCSI, duration of diabetes, metformin, sulfonylurea, TZD, DDP-4i, insulin basal, insulin premixed, insulin basal and bolus, number of oral hypoglycemic agents, ACEI/ARB, beta-blocker, CCB, diuretics, statin, fibrate, aspirin, and enrolled research year*.
